# The Role of Dual-Energy Computed Tomography in Locating Gastrointestinal Tract Perforations

**DOI:** 10.7759/cureus.15265

**Published:** 2021-05-27

**Authors:** Serap Baş, Elbrus Zarbaliyev

**Affiliations:** 1 Department of Radiology, Gaziosmanpaşa Hospital, İstanbul Yeni Yüzyıl University, İstanbul, TUR; 2 Department of General Surgery, Gaziosmanpaşa Hospital, İstanbul Yeni Yüzyıl University, Istanbul, TUR

**Keywords:** acute abdomen, gastrointestinal perforation, free air in the abdomen, dual-energy ct, emergency surgery

## Abstract

Objective

With each passing day, dual-energy computed tomography (DECT) is being used more frequently in the evaluation of abdominal pathologies. In this article, we aimed to assess the role of dual-energy CT in locating gastrointestinal perforations, which are among the causes of acute abdomen.

Materials and methods

All patients who underwent DECT due to acute abdomen in a COVID-19 designated hospital between June 1st, 2020 and December 31st, 2020, who were found to have gastrointestinal tract (GIT) perforation and underwent surgery were included in the study. DECT results and intraoperative findings of the patients were compared.

Results

Thirteen patients (nine males and four females) who underwent DECT for acute abdomen and were diagnosed with perforation in the gastrointestinal system were included in the study. The mean age of the patients was 57.6 years (range: 11-85 years). Two patients had gastric perforation, three had duodenal perforations, and one patient had a perforation in the gallbladder wall. Two patients were diagnosed with jejunal perforations, one patient with Meckel's diverticulum, and three patients with colorectal perforation. Although free air was detected in the abdomen of one patient, perforation could not be located. In patients with GIT perforation who were operated on following DECT imaging, the perforation location shown on DECT correlated 100% with the perforation locations detected during surgery.

Conclusion

DECT is significantly effective in planning surgical treatment and determining the foci of perforation in GIT perforations.

## Introduction

Gastrointestinal tract (GIT) perforations are still challenging clinical conditions to manage in surgical clinics. Particularly in non-traumatic perforations, the clinician's suspicion is crucial. Patient history, clinical course of the disease, physical examination, and different imaging techniques assist in showing the presence of GIT perforations. Despite this, it is not always possible to locate the perforation before surgery, and the exact location is determined intraoperatively. Although emergency surgical treatment is the standard treatment approach in GIT perforations, the localization of the perforation is very important in determining the surgical strategy, especially in the present pandemic conditions. Typically, these patients undergo preoperative abdominal tomography upon the detection of subdiaphragmatic free air by direct abdominal radiography in a standing position (DARS) [1]. The use of abdominal tomography is very common among patients with acute abdomen and is very useful in detecting possible pathologies and planning treatment [2]. In recent years, dual-energy computed tomography (DECT) has also been used for this purpose. Although DECT has been theoretically known since 1967, its clinical use started in 2006 [3-5]. DECT, which enables different tissues to be distinguished by scanning at varying energy levels, has upgraded conventional CT to the next level. DECT has become widespread in the evaluation of acute abdomen, though there are still few studies on the technique [4,6]. DECT offers various solutions to assess different GIT pathologies. Although studies examining the role of DECT in the evaluation of inflammatory and non-inflammatory diseases of GIT have been revealed in recent years, when we reviewed the literature, we could not find any research paper or study in which GIT perforations were examined and identified via DECT [7,8]. In this study, we aimed to investigate the role of DECT in determining the location of perforations in patients who developed GIT perforation for different reasons and in different regions.

## Materials and methods

The results of all patients who underwent DECT due to DARS and acute abdominal symptoms in a COVID-19 designated hospital between June 1st, 2020 and December 31st, 2020, were retrospectively included in the study. Patients who opted for non-operative intervention and did not accept surgery were not included in the study. The data related to the patients' age, sex, radiological results, and surgical findings were retrospectively reviewed, and the results of DECT and intraoperative surgery findings were compared, following the ethical approval of the Istanbul Yeni Yüzyıl University ethics committee, with the decision number 2021/02-586.

CT examination protocol

All patients underwent a multiphase abdominal contrast-enhanced dual-energy CT using a 160 mm, 3rd generation multidetector CT (MDCT) scanner (Revolution CT, GE Healthcare, Milwaukee, WI, USA). Firstly, 1.5 cc/kg of iodinated contrast medium (Visipaque 350 mg I/mL, GE Healthcare, Princeton, New Jersey) was injected intravenously through a power injector at a rate of 3 mL/sec to acquire a contrast-enhanced arterial and portal venous phase dual-energy CT. All phases included the whole abdomen, from the dome of the liver to the iliac crest within a one-breath hold. The arterial phase acquisition was launched four seconds after the attenuation increase of the abdominal aorta reached the predefined threshold of 120 HU (Smartprep, GE Healthcare, Waukesha-WI, USA), while the portal venous phase was launched 75 seconds after. Only the portal venous phase images were acquired in dual-energy mode. Arterial scanning mode had the following parameters: tube voltages 120 kV Assist mode (80-120), tube current SmartmA mode 100-400 mA, detector width 80 mm, helical pitch 0.992:1, rotation time 0.60 s, slice thickness 1.5 mm, and slice interval 1.5 mm. The portal venous phase, dual-energy scanning mode, had the following parameters: helical rapid switch between tube voltages of 80 and 140 kVp, tube current 320 mA, detector width 80 mm, helical pitch 0.992:1, rotation time 0.60 s, slice thickness 1.5 mm, and slice interval 1.5 mm.

Image reconstructions and analysis

All the images were reconstructed and processed using a dedicated workstation equipped with dual-energy CT post-processing software (AW 4.7, GE Healthcare, Milwaukee, WI, USA). The dual-energy CT reconstructions were virtual unenhanced, iodine overlay maps and virtual monochromatic imaging reconstructions. All images were archived to a picture archiving and communication system (PACS) for interpretation.

Firstly, the arterial phase and 120 kV mixed images of the portal venous phase were reviewed for free abdominal gas, which is the most important criteria for perforation. Then iodine overlay and virtual monoenergetic datasets were reviewed for the presence of necrosis of the GIT wall, which is determined with focal or diffuse transmural loss of wall enhancement on 120 kV simulated and 45 keV virtual monoenergetic images or transmural absence of iodine overlay images.

## Results

Surgical treatment was decided in 16 patients who underwent DECT due to acute abdomen. Two patients who were followed up nonoperatively for duodenal perforation and one patient who did not accept surgery despite the detection of colon diverticulum perforation were excluded from the study. Thirteen patients (nine males and four females) diagnosed with perforation in the gastrointestinal system were included (Table 1).

**Table 1:** Patient demographics and detected gastrointestinal tract perforation sites

Perforation	Patient age	Gender	Additional feature	Perforation site seen in DECT	Intraoperative perforation site	Surgery performed
Stomach	76	Female	Penetrating injury	Stomach small curvature	Stomach Lesser Curvature/antrum	Primary repair + omentoplasty
Stomach	58	Male	Hepatocellular carcinoma (HCC) + Acid	Stomach antrum	Stomach antrum	Primary repair + omentoplasty
Duodenum	79	Female	No	Duodenum	Duodenum	Primary repair
Duodenum	85	Female	Colon Hepatic Flexura Tumor	Duodenum	Duodenum	Partial resection + gastrojejunostomy
Duodenum	81	Female	After endoscopy	Duodenum 3rd Part	Duodenum 3rd Part	Primary repair
Gall bladder	65	Male	No	Gall bladder	Gall bladder	Laparoscopic cholecystectomy
Jejunum	74	Male	Lung cancer	Jejunum	Midjejunal region	Segmental resection
Jejunum	70	Male	Penetrating injury	Jejunum	Distal jejunum	Segmental resection
Sigmoid colon	16	Male	Systemıc Lupus Erythematosus ( SLE)	Sigmoid colon	Sigmoid colon	Primary suture and loop colostomy
Sigmoid colon	60	Male	Diverticulosis coli	Sigmoid colon	Sigmoid colon	Segmental resection
Rectosigmoid Anastomosis	43	Male	Sigmoid colon resection 3 Months Ago	Rectosigmoid Anastomosis	Rectosigmoid Anastomosis	Primary suture and loop colostomy
Free air is available in the abdomen	31	Female	Rectal cancer	Not found	Not found	Diagnostic laparotomy
Meckel's diverticulum	11	Male	No	Midileal	Meckel's diverticulum	Segmental resection

**Figure 1:**
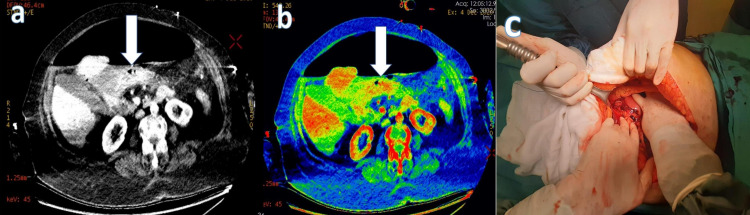
A 77-year-old female patient with abdominal pain, nausea, and vomiting had emergency DECT performed for the abdominopelvic region with IV contrast, and widespread free air in the abdomen and a perforation in the anterior stomach wall were detected. Transmural loss of wall enhancement on (a) 45 keV virtual monoenergetic image (white arrow) and (b) iodine overlay map images (white arrow). (c) Perforation localization at the junction of the small curvature antrum of the stomach.

The mean age of the patients was 57.6 years (range: 11-85 years). Upper GIT perforation was detected in five patients. Gastric perforation was detected in two of these patients, who had emergency surgery for primary repair (Figure 1).

**Figure 2:**
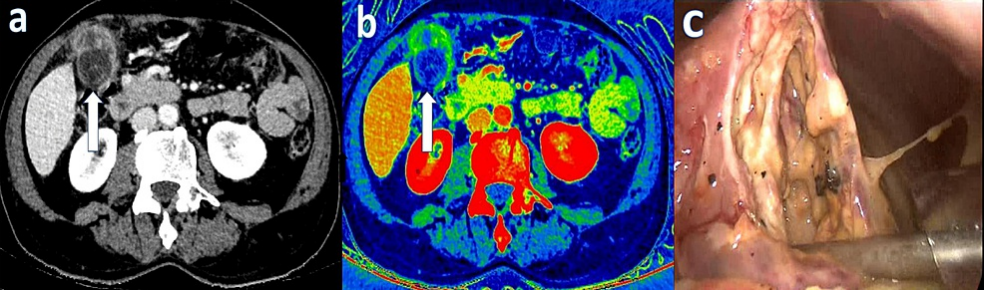
Dual-energy IV contrast abdominopelvic CT performed on a 66-year-old male admitted to the hospital with a complaint of pain in the right upper quadrant revealed perforation of the gallbladder wall. Transmural loss of wall enhancement on (a) 45 keV virtual monoenergetic images (white arrow) and (b) iodine overlay map images (white arrow). (c) Necrosis and perforation in the gallbladder wall.

In the other three patients, duodenal perforation was detected. First of all, primary repair and drainage surgery was performed in all of these patients for intra-abdominal sepsis findings in medical treatment plans and follow-up. A perforation was detected in the gallbladder of a patient, followed by laparoscopic cholecystectomy (Figure 2).

**Figure 3:**
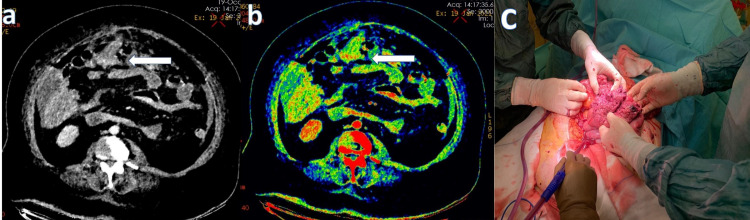
A 65-year-old female patient, who was operated on due to a malignant neoplasm of the corpus uteri, presented with abdominal pain and distension. In the IV contrast-enhanced dual-energy abdominopelvic CT examination, focal integrity loss in the small intestine in the midline of the abdomen and free air images were observed. Transmural loss of wall enhancement on (a) 45 keV virtual monoenergetic images (white arrow) and (b) iodine overlay map images (white arrow). (c) Perforation localization in the jejunal segment covered with necrotic tissue.

**Figure 4:**
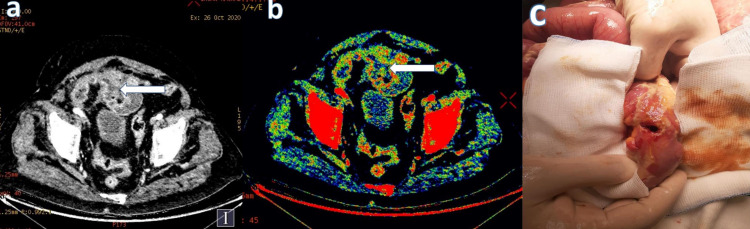
A 74-year-old male patient, who received radiotherapy to the pelvic region due to metastatic lung cancer, presented with abdominal pain and distension. In the dual-energy IV contrast abdominopelvic examination, loss of integrity in the distal ileal wall and free air in the abdominal cavity were observed. However, the contrast difference between the wall of the intestinal segment with disrupted wall integrity and the adjacent bowel loops was less than in other perforation patients. It was thought to be because of radiotherapy. Transmural loss of wall enhancement on (a) 45 keV virtual monoenergetic images (white arrow) and (b) iodine overlay map images (white arrow). (c) Perforation localization in the ileum after radiotherapy.

Perforation in the small intestine was detected in three patients. One patient had jejunum perforation, one patient each had jejunal, ileal, and Meckel`s diverticular perforations. Segmental resection and primary anastomosis were performed in all three patients (Figures 3, 4).

**Figure 5:**
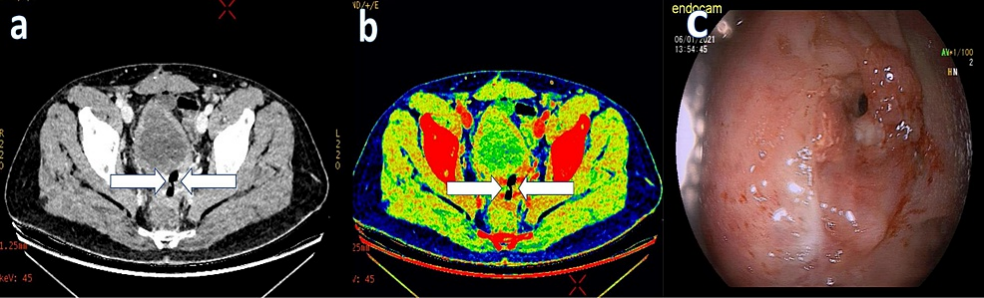
A 43-year-old male patient, who was operated on for rectal cancer, showed loss of integrity in the rectosigmoid anastomosis line and free air in its neighborhood in the IV contrast-enhanced abdominopelvic CT performed with DECT. It was considered a closed perforation. Transmural loss of wall enhancement on (a) 45 keV virtual monoenergetic images (white arrow) and (b) iodine overlay map images (white arrow). (c) Late anastomotic leak in the rectosigmoid anastomosis line.

Colorectal perforation was reported in three patients. Segmental resection and anastomosis were performed in these patients with sigmoid diverticulum perforation. In other patients, loop colostomy was performed with primary repair (Figure 5).

**Figure 6:**
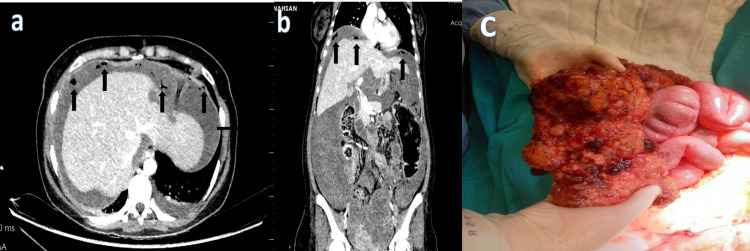
A 31-year-old female patient treated for metastatic colon cancer developed abdominal pain and distension, and free air (black arrows) was observed in the perihepatic area on dual-energy abdominopelvic CT with IV contrast. Perforation focus was not detected on CT examination. (a) Axial image. (b) Coronal image. (c) No perforation was found in the patient who underwent laparotomy due to free air in the abdomen.

In one patient, although free air was detected in the abdomen, the localization of the perforation could not be determined (Figure 6).

Appropriate surgical treatment was administered to all patients, considering the pathologies and intraoperative findings indicated as a result of DECT. There was no mortality except one patient who developed duodenal perforation.

In cases where laparotomy was required, only upper midline (UM) or lower midline (LM) incision, which is suitable for the stated pathology, was performed instead of the standard UM + LM (upper midline + lower midline) incision, which is often preferred during acute abdomen due to perforation. No patient required additional incision enlargement. In all patients, it was observed that the perforation locations detected intraoperatively corresponded to the localizations specified in the result of DECT that was performed before surgery. In one patient whose perforation location could not be determined by DECT, foci of intraoperative perforation could not be found.

## Discussion

In emergency surgery clinics, GIT perforations remain a serious health problem as a cause of acute abdomen. Albeit treatment* *with proton pump inhibitors (PPI) results in a significant decrease in upper GIT perforations due to peptic ulcer, traumatic and nontraumatic perforations are still quite common [9]. Due to the severity of the clinical manifestation, rapid diagnosis and treatment are vital in these patients [10]. Although surgery is the primary and often the only option in the treatment, numerous radiological examination alternatives are available for diagnosis [1]. Direct abdominal radiographs have been used as the first option for diagnosis for a long time; however, GIT perforations have increasingly been diagnosed with conventional CT with or without contrast over time [10,11]. The widespread use of CT speeds up the surgical treatment in these patients and helps determine the correct strategies. Contrast shows 82-90% accuracy, depending on the route of use (intravenous, oral) [1,2,12]. Determination of regional ischemia and necrosis in nontraumatic GIT perforations, extravasation of oral contrast, and determination of the presence of regional free air allow localization with 93.3% sensitivity and 95.9% specificity [12]. DECT carries this advantage of conventional CT even further by eliminating the need for oral contrast, not requiring multiple exposure phases, precisely determining ischemic/necrotic areas that may cause perforation using different energy levels, and showing the presence of additional pathologies in detail [4,8]. Moreover, a 14-34% reduction in radiation dose is considered as another remarkable advantage [13,14]. In our healthcare center, all patients who presented with acute abdomen for the last year have been evaluated via DECT imaging. It is aimed at determining wall necrosis and potential perforation sites in patients with GIT perforation by reconstructing virtual monoenergetic images (VMIs) and iodine overlay images.

This employed method was determined to be effective in all luminal structures of the gastrointestinal system. DECT is becoming more and more common in the evaluation of stomach pathologies [15,16]. Particularly, studies assessing the size of gastric tumors and their local response following neoadjuvant therapy have been reported. Ischemic lesions are less common due to gastric hyperemia [17]. Peptic ulcer perforations are exceptional, and local ischemic foci are seen in the stomach wall [18]. Through the reconstruction of VMIs, we detected that the widespread free air in the abdomen of two patients who developed gastric perforation originated from the small curvature of the stomach. The patients were operated on, and primary repair was performed through UM incision. Isolated small bowel perforations are rare and often develop after penetrating ileus, mesenteric ischemia, traumas, malignancies, and rarely after radiotherapy [19]. In the literature, evaluation studies of DECT, which involve small bowel pathologies, are increasing day by day [4,20,21]. It was underscored in the study of Gosangi et al., which examined the radiological findings of duodenal pathologies, that DECT could be a good alternative [22]. In our study, there were three patients with duodenal perforation. Oral non-contrast DECT imaging was performed in these three patients for acute abdomen, and images were processed to determine the foci of perforation. Local ischemia and perforation sites were detected in the duodenum of all three patients through reconstruction of VMIs. Duodenal repair surgery of patients in the early period was performed through UM incision.

In their study investigating the role of DECT in GIT pathologies, Murray et al. suggested that the method was remarkably effective in detecting ischemic areas in GIT [6]. Therefore, it has been highlighted that it could be useful in assessing possible perforation areas. However, no information was found in the literature related to the exemplary cases. Similar localization studies were performed in the jejunal and ileal segments via the employed method, and it was observed that DECT and surgical findings were compatible in the other three patients as well. We determined that in one of our patients who developed GIT perforation due to radiotherapy, the contrast enhancement difference between the perforated segment and the adjacent intestinal wall was lesser at a low keV value (45 keV) compared to other patients. This lower difference could be due to radiotherapy-induced vasculopathy and insufficient intestinal wall nutrition.

The role of DECT in the investigation of colorectal pathologies has been increasing for the last 10 years. In 2009, Karaaltıncaba et al. reported the results of their virtual colonoscopy, which was performed through DECT [23]. Besides, in their studies, Yang et al. demonstrated the role of DECT in the detection of colorectal lesions [24]. Moreover, Elbanna et al. have revealed that DECT is superior in patients with gangrenous appendicitis [25]. However, no clinical studies investigating the role of DECT in colorectal perforations have been reported in the literature. Colorectal perforations due to varying reasons are more common among the community at advanced ages [26]. Albeit it generally occurs because of malignant tumors and diverticular disease of the small bowel, it can also occur due to other reasons (such as trauma or anastomotic leaks). In our study, a patient was detected with anastomotic leak and the development of spontaneous diverticulum perforation in the colon, and the locating perforation after the DECT was performed in all patients without the administration of oral contrast. To the best of our knowledge, these colorectal cases in our study are the first in terms of using DECT in patients with colorectal perforation and anastomotic leak.

Ultrasonography is the first preferred radiological examination in the assessment of biliary system pathologies [27]. Patients generally present to the emergency services with acute abdomen because of perforation that develops in the advanced stages of acute pathologies of the gallstone. As in all patients with acute abdomen, these patients are assessed via conventional CT imaging. However, the evaluation of different gallbladder pathologies with conventional CT has some limitations (such as gallstone features, gallbladder wall structure) [6]. Due to these limitations, DECT has been on the agenda frequently in recent years [28]. By evaluating the properties of the tissues at different keV values, Ratanaprasatporn et al. revealed that the gallbladder pathologies were examined in more detail by DECT than conventional CT [29]. Particularly, inflammation of the gallbladder wall and deterioration of the integrity of the gallbladder wall in later stages can be seen more clearly [4,6,28]. Murray et al. underscored that this feature is a remarkable finding in gallbladder perforations [6]. In one of the patients in our study, gall bladder perforation was detected in low keV values after DECT imaging, which was performed due to acute abdomen, and laparoscopic cholecystectomy was performed on the patient.

The presence of free air in the abdomen, which was detected by imaging methods in patients presenting with acute abdomen, is a significant finding that causes suspicion of GIT perforation and should be considered [30]. In non-traumatic patients, the occurrence of free air in the abdomen, with a rate of 5-10%, is not accompanied by intrabdominal perforations. Diagnosis is mostly made after laparotomy due to the lack of causation, and additional morbidity is loaded on the patient after this surgical procedure. Hence, detection of idiopathic free air in the abdomen in advance is crucial for treatment planning. Through DECT imaging, we detected free air in the abdomen of a patient who was scheduled for chemotherapy due to metastatic rectum and development of acute abdomen because of ileus. After assessing different keV values, the patient, whose perforation foci could not be detected, was operated on. No foci of perforation were found in the exploration either.

Our study is the first study in the literature in terms of using DECT and conducting surgical correlation in GIT perforations. The low number of patients included in the study and the inhomogeneity of the patient group can be considered as a remarkable limitation of our study. Thus, it is evident that further studies are needed to investigate the role of DECT in locating GIT perforations.

## Conclusions

In patients with GIT perforation, who were taken into operation (due to widespread intraabdominal infection and impaired general condition) following DECT imaging, the perforation localization shown on DECT correlated with the perforation localization detected during surgery. Thus, we consider that DECT is a significantly effective and reliable method in revealing perforation localization. However, this result may not be accurate due to the small inhomogeneous patient group. Therefore, conducting multi-center studies involving a large number of patients will be beneficial in obtaining more accurate proportions.

## References

[1] Shin D, Rahimi H, Haroon S, Merritt A, Vemula A, Noronha A, LeBedis CA (2020). Imaging of gastrointestinal tract perforation. Radiol Clin North Am.

[2] Kloth C, Vogele D, Brunner H, Beer M, Schmidt SA (2020). Pathognomonic imaging signs in abdominal radiology. Abdom Radiol (NY).

[3] Genant HK, Boyd D (1977). Quantitative bone mineral analysis using dual energy computed tomography. Invest Radiol.

[4] Silva AC, Morse BG, Hara AK, Paden RG, Hongo N, Pavlicek W (2011). Dual-energy (spectral) CT: applications in abdominal imaging. Radiographics.

[5] Johnson TR, Krauss B, Sedlmair M (2007). Material differentiation by dual energy CT: initial experience. Eur Radiol.

[6] Murray N, Darras KE, Walstra FE, Mohammed MF, McLaughlin PD, Nicolaou S (2019). Dual-energy CT in evaluation of the acute abdomen. Radiographics.

[7] (2013). Diagnostic Radiology: Recent Advances and Applied Physics in Imaging.

[8] Trabzonlu TA, Mozaffary A, Kim D, Yaghmai V (2020). Dual-energy CT evaluation of gastrointestinal bleeding. Abdom Radiol (NY).

[9] Malviya A, Hussain A, Bulchandani HP, Bhardwaj G, Kataria S (2017). A comprehensive study on acute non-traumatic abdominal emergencies. Int J Surg.

[10] Patterson JW, Kashyap S, Dominique E (2020). Acute abdomen. StatPearls [Internet].

[11] Zeina AR, Shapira-Rootman M, Mahamid A, Ashkar J, Abu-Mouch S, Nachtigal A (2015). Role of plain abdominal radiographs in the evaluation of patients with non-traumatic abdominal pain. Isr Med Assoc J.

[12] Menke J (2010). Diagnostic accuracy of multidetector CT in acute mesenteric ischemia: systematic review and meta-analysis. Radiology.

[13] Henzler T, Fink C, Schoenberg SO, Schoepf UJ (2012). Dual-energy CT: radiation dose aspects. AJR Am J Roentgenol.

[14] Zhang LJ, Peng J, Wu SY (2010). Liver virtual non-enhanced CT with dual-source, dual-energy CT: a preliminary study. Eur Radiol.

[15] Pan Z, Pang L, Ding B (2013). Gastric cancer staging with dual energy spectral CT imaging. PLoS One.

[16] Liang P, Ren XC, Gao JB, Chen KS, Xu X (2017). Iodine concentration in spectral CT: assessment of prognostic determinants in patients with gastric adenocarcinoma. AJR Am J Roentgenol.

[17] Lefkovitz Z, Cappell MS, Kaplan M, Mitty H, Gerard P (2000). Radiology in the diagnosis and therapy of gastrointestinal bleeding. Gastroenterol Clin North Am.

[18] Malfertheiner P, Schulz C (2020). Peptic ulcer: chapter closed?. Dig Dis.

[19] Dal F, Topal U, Sözüer EM, Arıkan TB, Bozkurt GK, Akyüz M (2021). Our surgical experience and clinical results in non-traumatic small bowel perforations. Turk J Colorectal Dis.

[20] Mileto A, Ananthakrishnan L, Morgan DE, Yeh BM, Marin D, Kambadakone AR (2020). Clinical implementation of dual-energy CT for gastrointestinal imaging. (IN PRESS). AJR Am J Roentgenol.

[21] Guler E, Unal NG, Hekimsoy I, Kose T, Harman M, Ozutemiz AO, Elmas NZ (2021). Dual-energy CT enterography in evaluation of Crohn's disease: the role of virtual monochromatic images. Jpn J Radiol.

[22] Gosangi B, Rocha TC, Duran-Mendicuti A (2020). Imaging spectrum of duodenal emergencies. Radiographics.

[23] Karcaaltincaba M, Karaosmanoglu D, Akata D, Sentürk S, Ozmen M, Alibek S (2009). Dual energy virtual CT colonoscopy with dual source computed tomography: initial experience. Rofo.

[24] Yang Z, Zhang X, Fang M, Li G, Duan X, Mao J, Shen J (2019). Preoperative diagnosis of regional lymph node metastasis of colorectal cancer with quantitative parameters from dual-energy CT. AJR Am J Roentgenol.

[25] Elbanna KY, Mohammed MF, Chahal T, Khosa F, Ali IT, Berger FH, Nicolaou S (2018). Dual-energy CT in differentiating nonperforated gangrenous appendicitis from uncomplicated appendicitis. AJR Am J Roentgenol.

[26] Yaman İ, Kara C, Karabuğa T, Sözütek A, Tansuğ T, Bozdağ AD, Nazlı O (2010). Clinical evaluation and treatment results of patients with nontraumatic colon perforation. Turk J Colorectal Dis.

[27] Gandolfi L, Torresan F, Solmi L, Puccetti A (2003). The role of ultrasound in biliary and pancreatic diseases. Eur J Ultrasound.

[28] Matos C (2019). Will dual-energy CT become the reference standard to evaluate gallstone disease?. Radiology.

[29] Ratanaprasatporn L, Uyeda JW, Wortman JR, Richardson I, Sodickson AD (2018). Multimodality imaging, including dual-energy CT, in the evaluation of gallbladder disease. Radiographics.

[30] Taubmann O, Li J, Denzinger F, Eibenberger E, Müller FC, Brejnebøl MW, Maier A (2020). Automatic detection of free intra-abdominal air in computed tomography. Medical Image Computing and Computer Assisted Intervention - MICCAI 2020. MICCAI 2020. Lecture Notes in Computer Science.

